# Process variations between Swiss units treating neonates with hypoxic-ischemic encephalopathy and their effect on short-term outcome

**DOI:** 10.1038/s41372-021-01156-w

**Published:** 2021-07-21

**Authors:** Mark Adams, Barbara Brotschi, André Birkenmaier, Katharina Schwendener, Verena Rathke, Michael Kleber, Cornelia Hagmann, Swiss National Asphyxia and Cooling Register Group, Dirk Bassler, Giancarlo Natalucci, Susanne Böttger, Bernhard Frey, Vera Bernet, Beate Grass, Bjarte Rogdo, Irene Hoigné, Martin Stocker, Thomas M. Berger, Matteo Fontana, Lukas Hegi, Philipp Meyer, Gabriel Konetzny, Sven M. Schulzke, Sven Wellmann, Maya Hug, Tilman Humpl, Bendicht Wagner, Karin Daetwyler, Thomas Riedel, Brigitte Scharrer, Nicolas Binz, Anita Truttmann, Juliane Schneider

**Affiliations:** 1 Newborn Research, Department of Neonatology, University and University Hospital Zurich, Zurich, Switzerland; 2 Division of Neonatology and Pediatric Intensive Care, Children’s University Hospital Zurich, Zurich, Switzerland; 3 Department of Neonatology and Pediatric Intensive Care, Children’s Hospital St. Gallen, Neonatal and Pediatric Intensive Care Unit, St. Gallen, Switzerland; 4 Department of Neonatology and Pediatric Intensive Care, Children’s Hospital, Spitalstrasse, Lucerne, Switzerland; 5 Clinic of Neonatology, Cantonal Hospital Winterthur, Winterthur, Switzerland; 6 Department of Neonatology, Children’s Clinic, Cantonal Hospital Aarau, Aarau, Switzerland; 7 Department of Neonatology, University Children’s Hospital Basel (UKBB), Basel, Switzerland; 8 Department of Pediatric Intensive Care, University Hospital Berne, Berne, Switzerland; 9 Department of Neonatology, Children’s Hospital Chur, Chur, Switzerland; 10 Department of Neonatology, University Hospital (CHUV), Lausanne, Switzerland

**Keywords:** Translational research, Outcomes research, Health services

## Abstract

**Objective:**

To compare therapeutic hypothermia (TH) treatment of term and near-term neonates with hypoxic-ischemic encephalopathy (HIE) between neonatal units.

**Study design:**

Population-based, retrospective analysis of TH initiation and maintenance, and of diagnostic imaging. The comparison between units was based on crude data analysis, indirect standardization, and adjusted logistic regression.

**Results:**

TH was provided to 570 neonates with HIE between 2011 and 2018 in 10 Swiss units. We excluded 121 off-protocol cooled neonates to avoid selection bias. Of the remaining 449 neonates, the outcome was favorable to international benchmarks, but there were large unit-to-unit variations in baseline perinatal data and TH management. A total of 5% neonates did not reach target temperature within 7 h (3–10% between units), and 29% experienced over- or undercooling (0–38%).

**Conclusion:**

Although the neonates had favorable short-term outcomes, areas for improvement remain for Swiss units in both process and outcome measures.

## Introduction

At the turn of the millennia, therapeutic hypothermia (TH) became standard of care for neonates with moderate-to-severe hypoxic–ischemic encephalopathy (HIE) in high-income countries [1–4]. Systematic reviews of cooling trials showed that TH was able to reduce the combined outcome of death or major neurodevelopmental disability in survivors up to childhood, with a number needed to treat of 7 for an additional beneficial outcome [2]. Long-term impairment among survivors still remains an important issue [5]. Trials examining whether pharmacological or nonpharmacological interventions in combination with TH augment the neuroprotective effect of TH are in progress. Until such add-on therapies are available, it is essential to further optimize the clinical management for TH as evidence suggests that current cooling protocols for 72 h are reasonably close to optimal [6].

In 2011, Swiss neonatal units agreed on a cooling protocol for standardized treatment of HIE based on those previously used for randomized controlled trials (RCTs) [1]. However, RCTs have well-defined protocols and independent data monitoring committees to monitor the progress of a clinical trial, safety data, and critical efficacy variables to guarantee conduction according to the protocol. Such a thorough process cannot be upheld in an everyday clinical setting. Real-world data are collected outside the controlled restrictions of RCTs and are therefore more representative of usual clinical practice [7]. In order to monitor neonates with HIE and provide real-world outcome data, the affected neonates were since registered in the Swiss National Asphyxia and Cooling Register [8]. Several benefits of maintaining a register for neonates with HIE have since been discussed [9, 10]. A retrospective comparison between neonates treated with TH before and after the implementation of the Swiss register revealed an overall improved management with reduced temperature variability, more comprehensive improved neuromonitoring, and higher follow-up rates [11]. A possible effect of treatment variations on outcome was not investigated.

In this study, we analyze the adherence of the Swiss neonatal units to the agreed standard TH protocol for all cooled neonates between 2011 and 2018. We compared the resulting variation of protocol deviations between units. To the involved units, this provides feedback toward quality-improvement potential. To everyone else, it displays where difficulties lied in implementing TH in a real-world setting in contrast to those of RCTs. In a second step, we test for the existence of known associations between process deviations and short-term outcome in our collective to measure the effect those deviations may have had on the collective.

## Methods

Data collection, evaluation, and publication for this study was approved by the Swiss ethical committee and the Swiss Federal Commission for Privacy Protection in Medical Research (KEK-ZH-Nr2014-0551 and KEK-ZH-Nr2014-0552). Participating centers were obliged to inform parents about the scientific use of anonymized data.

### Study population

This was a population-based retrospective cohort study of prospectively collected data on all Swiss neonates receiving TH between 2011 and 2018 (*N* = 578). Data of one unit contributing eight neonates had to be excluded due to incompleteness of data. All other units complied to data plausibility and completeness checks and annual data audits were performed by the study coordinator (VR). Data collection was performed at the National Asphyxia and Cooling Register hosted by SwissNeoNet.

### National protocol for standardized treatment of HIE

The standardized Swiss protocol (SSP) for treatment of HIE with TH was implemented in 2011 (Supplement 1). It summarizes eligibility for TH, target temperature, initiation and duration of TH, clinical management/neuromonitoring, and neuroimaging during and after TH. It was based on the results from basic research, protocols used for TH in RCTs, and reviews on diagnostics [1, 12–17].

### Perinatal characteristics and neurological assessment

Demographical maternal, pregnancy, delivery, and neonatal data were extracted from the national register. The severity of HIE was measured by a neurological examination using Sarnat or Thompson assessments. Experienced neonatologist performed the examinations on admission prior to cooling, and thereafter daily until rewarming was finished. In order to compare severity of HIE on admission between centers, Thompson scores (used by one center) were converted to Sarnat scores (used by all other centers): Thompson scores 1–6 to Sarnat score 1, Thompson scores 7–12 to Sarnat score 2, and Thompson scores 13–22 to Sarnat score 3, respectively [13, 18].

### Process–quality indicators (QI)

The following processes defined by the SSP were analyzed for unit-specific deviation from the protocol:

The term “*off-protocol cooling*” refers to neonates not fulfilling the inclusion criteria for TH according to the SSP. This includes neonates with mild HIE (Sarnat score = 1), neonates <35 weeks’ gestational age at birth, neonates with life-threatening major congenital malformations, postnatal age at the onset of TH > 6 h, or neonates that had neither of the following clinical or biochemical conditions: an Apgar score < = 5 at 5 or 10 min, a continued need for resuscitation, or a pH < = 7.0, and a base deficit > = 16 mmol/L or lactate > = 12 mmol/L in umbilical cord or blood sample with 60 min after birth.

The protocol further instructs that TH should be started as soon as practically possible, but within the first 6 h after birth. Target temperature of 33.0–34.0 °C should be reached as soon as possible, optimally within one to 2 h after initiation of hypothermia and maintained for 72 h. Hourly temperature-point measures of core temperature by rectal probe were recorded. We monitored all incidences where *target temperature was not reached within 7* h and defined this as a quality indicator. We monitored whether the target temperature was maintained within range. To capture large deviations and avoid random noise, *over- or undercooling* during TH was defined as temperature being outside the target temperature for 3 consecutive hours during TH. The SSP targets rewarm at a rate of no more than 0.2–0.5 °C per hour. We therefore monitored incidences where *rewarming was* >*0.5* *°C per hour* using an algorithm to capture the time points when a temperature <34.0 °C was measured for the last time and when 36.0 °C or above was recorded a first time.

We further monitored processes known to improve diagnostics and prediction of outcome, i.e., whether a *cerebral ultrasound (cUS) was performed on admission* and whether magnetic resonance imaging (*MRI) was performed between days 5 and 14 of life*, i.e., after termination of TH.

Although not specified by the SSP, we also analyzed the effect of passive versus active cooling, as active cooling had shown greater temperature stability in a prior publication of our group and centers had since gradually changed to active cooling [11].

### Outcome variables

The investigated short-term outcome QIs were arterial hypotension (defined as “hypotension requiring treatment as defined by unit policies” during day 1–4), seizures (defined as “clinical or subclinical seizures identified on amplitude-integrated electroencephalogram during TH and rewarming), infections (consisting of culture-proven sepsis or necrotizing enterocolitis according to Bell stages 2 and 3 [19]), coagulopathy (any disorder requiring treatment in order to maintain or recover normal hemostasis according to unit’s policy), persistent pulmonary hypertension (PPHN, severe hypoxemia disproportionate to the severity of lung disease, evidence of a right-to-left shunt and other findings suggesting PPHN in echocardiography and the need for medication), and mortality.

### Statistical methods

Descriptive analysis of crude process QIs as well as indirectly standardized unit-to-unit comparison using the entire pool of Swiss data as reference population (=1) and 95% confidence intervals. We imputed missing data (based on plausibility) as follows: of 10 neonates with unknown survival status, 1 was determined as died due to a diagnosis of multiple-organ failure, 4 were determined survivors due to complete 95 h temperature monitoring and discharge-planning data, and 5 as survivors because of the available 2 year follow-up data. A neonate with unknown birth location was determined outborn based on place of birth.

To determine the quality indicators for TH start, over-/undercooling, and rewarming, we used algorithms that scanned the hourly temperature measurements recorded from hours 0 to 95 as of cooling start. The algorithm required valid hourly temperature measurements per patient of at least 80% of all temperatures during hours 0–11 for TH start, hours 0–71 for over-/undercooling during TH and hours 60–84 for rewarming at the end of TH to determine when cooling started, where it varied over time, when it ended, and how fast rewarming occurred. For each resulting QI, data completeness of 90% of valid patient responses was required per cooling center. Less than 90% data completeness was determined unreliable and not evaluated.

Logistic regression was performed to analyze the associations between process QIs and selected outcome measures. Adjustment for indirect standardization and logistic regression was based on male sex, small for gestational age, Sarnat score on admission, composite pregnancy complication (maternal diabetes, maternal fever, or pre-eclampsia), composite-delivery sentinel events (placental abruption, ruptured uterus, shoulder dystocia, cord mishaps, or head entrapment), and being outborn. All analyses were performed in R version 3.6.1 [20].

## Results

### Study population

**Fig. 1:**
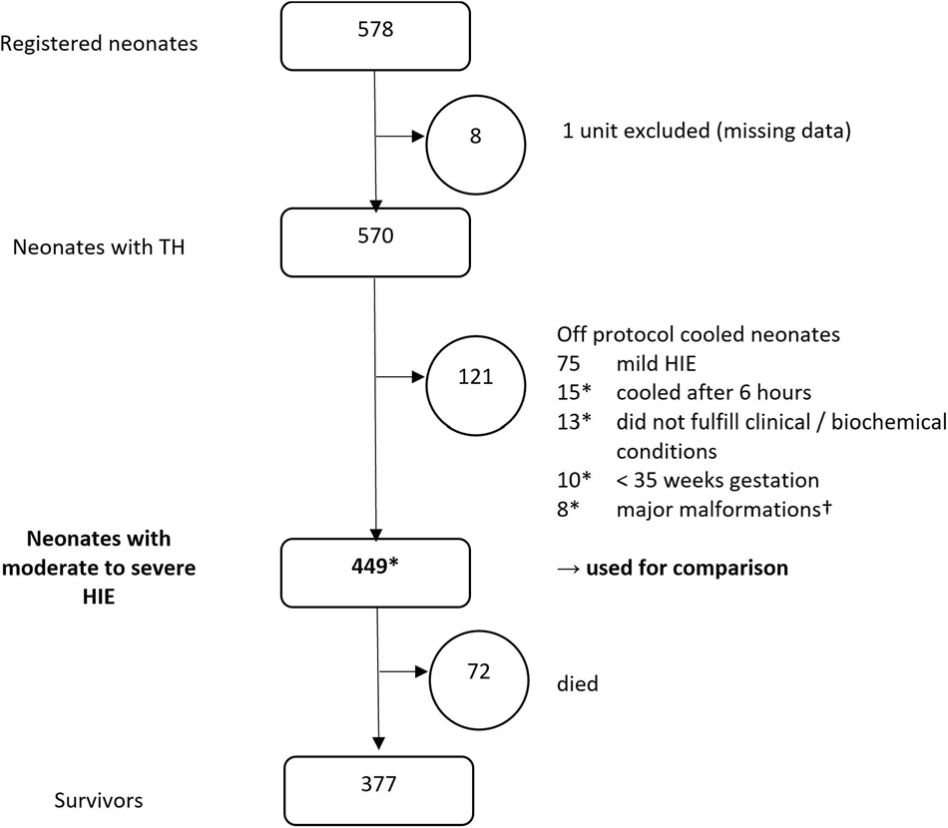
Study flow chart. *Estimated total number of neonates with moderate to severe HIE in Switzerland 2011-2018: 449 + 45 (missing unit of average unit size) + 46 (off-protocol cooled neonates with moderate-to-severe HIE) = 540, i.e., ca. 68 per year. † major malformations: 2 trisomy 21, 1 congenital diaphragmatic hernia, 1 transposition of the large vessels, 1 esophageal atresia, 1 hydrops fetalis, 1 Turner syndrome, 1 microcephalia. HIE: hypoxic ischemic encephalopathy; TH: therapeutic hypothermia.

The Swiss National Asphyxia and Cooling Register registered 578 cooled neonates between 2011 and 2018 (Fig. 1). Data from 1 unit (*n* = 8) was excluded due to incompleteness.

Of the 570 neonates receiving TH in the remaining 10 units, 118 were “off-protocol cooled neonates” (Table 1): 73 had mild HIE (Sarnat score of 1), 15 were cooled after 6 h of postnatal age, 13 did not fulfill specified clinical/biochemical conditions, 10 were <35 weeks’ gestation, and 7 had major malformations (Fig. 1). As they introduce bias into the comparison between units and with international data, all further analyses (including baseline characteristic analysis) were based on the dataset of 449 neonates fulfilling the inclusion criteria of the SSP.

**Table 1:** Unit specific baseline characteristics.

	Unit 1	Unit 2	Unit 3	Unit 4	Unit 5	Unit 6	Unit 7	Unit 8	Unit 9	Unit 10	Total
N cooled neonates	107	53	72	30	128	14	50	29	41	46	570
Off-protocol cooled neonates *N* (%)	38 (36%)	7 (13%)	9 (13%)	4 (13%)	25 (20%)	3 (21%)	9 (18%)	7 (24%)	7 (17%)	12 (26%)	121 (21%)
**N for unit to unit comparison**	**69**	**46**	**63**	**26**	**103**	**11**	**41**	**22**	**34**	**34**	**449**
Gestational age, median (iqr)	39 (38–41)	39 (38–40)	40 (39–41)	39 (37– 40)	40 (38–41)	40 (39–41)	40 (38–41)	39 (38–41)	40 (38–41)	40 (39–41)	40 (38–41)
Birth weight < 3rd percentile in %	7	2	0	0	5	9	2	0	9	6	4
Male sex in %	64	57	49	54	58	73	46	50	65	59	57
Sarnat score 3 at admission in %	10	52	37	35	32	0	10	41	24	24	28
Pregnancy complications^a^ in %	6	7	16	12	11	27	17	14	9	6	11
Delivery sentinel events^†^ in %	28	41	30	50	38	64	41	36	47	32	37
Pathological CTG in %	65	48	32	35	36	36	29	36	47	59	43
Emergency C section in %	57	54	35	42	32	36	40	36	47	6	39
Outborn in %	77	63	97	73	83	36	68	64	79	56	76
Apgar 1 min, median (iqr)	1 (0–2)	1 (0–2)	1 (1–3)	1 (0–1)	1 (0–2)	1 (1–2)	1 (0–2)	1 (0–2)	1 (0–3)	1 (0–3)	1 (0–2)
Apgar 10 min, median (iqr)	5 (3–6)	4 (2–6)	4 (2–6)	3 (2–4)	4 (3–7)	5 (4–6)	4 (4–6)	5 (4–6)	4 (3–6)	3 (2–5)	4 (3–6)
Resuscitated >10 min in %	71	74	67	85	44	55	51	68	62	62	61
Umbilical artery pH < = 7.0 in %	73	68	55	65	58	56	70	52	43	39	59
Base deficit > = 16 mmol/L in %	46	38	75	35	6	36	0	12	45	10	31
Head circ. at birth obtained in %	100	96	60	88	77	100	93	95	97	97	87

*C-section* caesarean section, *CTG* cardiotocography,* head circ.* head circumference, *iqr* interquartile range, *N* number of neonates.

^a^Pregnancy complications: diabetes, preeclampsia, maternal fever.

^†^Delivery sentinel events: placental abruption, ruptured uterus, shoulder dystocia, cord mishaps, or head entrapment.

Based on the 449 neonates receiving TH plus an estimated missing 45 neonates from the excluded unit that was of average size, plus the 46 neonates that were diagnosed with moderate-to-severe HIE but were treated off-protocol, we estimate a total of 540 cases of moderate to severe HIE in Switzerland between 2011 and 2018. With 680,664 live births during the study period, this yields a current period prevalence of ~0.8 per 1000 livebirths in Switzerland.

### Baseline characteristics

Considerable unit-specific variation in baseline pregnancy, maternal, delivery, and neonatal characteristics of the 449 neonates is shown in Table 1. The rates of outborns varied between 36% and 100% (neonatal unit based at a Children’s University Hospital with no labor ward). Pregnancy complications ranged from 6 to 27%, delivery sentinel events from 28 to 64%, birth weight was below the 3rd percentile in 0–9%, umbilical artery pH was below 7.0 in 39–73% and base deficit was above 16 mmol/L in 0–75% of cases. To account for their effect in the comparison between units, these parameters were adjusted for in the comparison of processes and short-term outcome.

### Processes

**Fig. 2:**
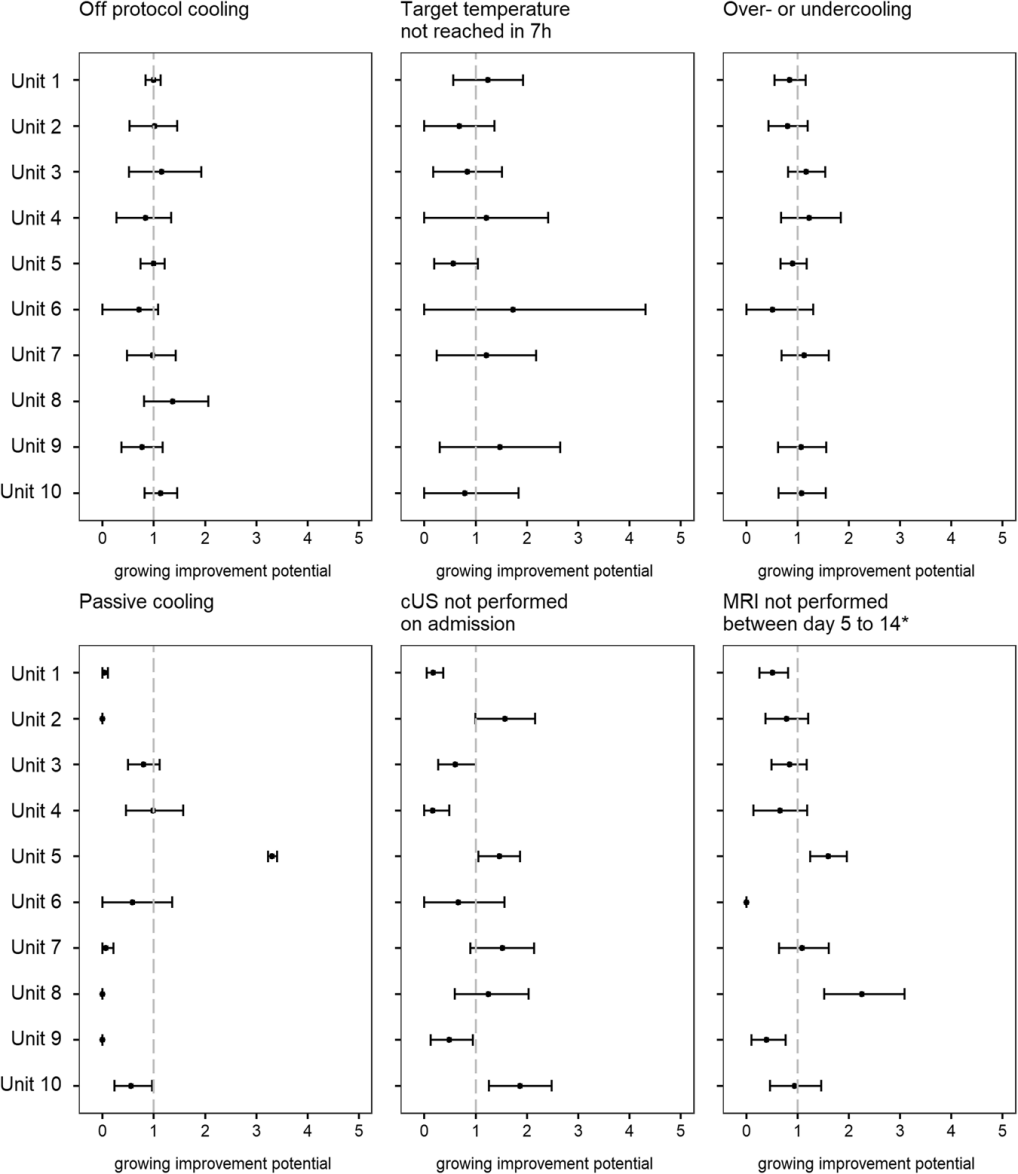
Unit specific standardized mortality/morbidity ratio overview with 95% confidence intervals of process deviations. Standardized, risk-adjusted processes as observed over expected ratios with the entire cohort as reference (=1). *analysis of survivors only.

The high variation in baseline characteristics is mirrored in how the units adhered to the SSP. For instance, neonates were cooled off-protocol between 9 and 36% of cases (Table 1). Quality indicators based on temperature measurements were deemed reliable only when data completeness requirements were met. The first three rows of Table 2 display the proportions of incomplete data per unit per algorithm used. All measurements for unit 8 as well as most measurements involving rewarming were missing in more than 10% of neonates and were therefore deemed as unreliable for this study. Of the remaining data, variation between units was well observable regarding mean time to reach target temperature that ranged from 3.5 to 4.5 h and subsequently target cooling temperature not being reached within 7 h in 3–10%, over- or undercooling occurred in 0–38%, passive cooling in 0–100%, no cUS was performed on admission in 4–47% and no MRI was performed after cooling in 0–35%. Figure 2 displays the standardized, risk-adjusted processes as observed over expected ratios with 95% confidence intervals with the entire cohort as reference (=1). Unreliable results were excluded. The above observed crude unit-to-unit variations are less prominent after standardization. However, some units display improvement potential when point estimates lie on the right-hand side of the reference value, particularly if confidence intervals do not overlap 1:. more-than-expected neonates did not reach target temperature within 7 h in units 1, 4, 6, 7, and 9; or they experienced over- or undercooling in units 3, 4, and 7; or they were more often cooled passively in unit 5; and cUS was not performed on admission in more-than-expected neonates in units 2, 5, 7, and 10. The large confidence intervals displayed for target temperature not reached in 7 h may reflect the lack of predictive validity of the risk-adjustment variables used, i.e., the lack of association between the risk factors and the neonates being cooled too late.

**Table 2:** Unit specific incomplete data recording, process deviations and short-term outcome.

	Unit 1	Unit 2	Unit 3	Unit 4	Unit 5	Unit 6	Unit 7	Unit 8	Unit 9	Unit 10	Total
Temperature recording incomplete between 0 and 11 h in %	0	6	2	0	1	9	0	23	3	3	3
Temperature recording incomplete between 0 and 71 h in %	2	6	3	0	3	9	0	18	0	0	3
Temperature recording incomplete between 60 and 84 h in %	42	28	20	21	26	9	8	39	10	42	26
Time to reach target temperature (h), mean (sd)	4.3 (2)	3.6 (1.9)	4.3 (2.3)	4.1 (2.1)	4.1 (1.8)	4.5 (2.2)	4.2 (2.2)	4.9 (2)	3.8 (1.8)	3.5 (1.7)	4.1 (2)
Target temperature not reached in 7 h in %	7.2	2.3	4.8	3.8	3.9	10	9.8	*11.8*	3	0	5
aEEG not performed daily during cooling and rewarming in %	13	60.9	17.5	15.4	32	18.2	68.3	*31.8*	17.6	17.6	29.8
Temperature on admission in °C, mean (sd)	34.4 (1.6)	34.5 (1.5)	34.8 (1.6)	34.9 (1.5)	34.9 (1.5)	35.1 (1.2)	35.7 (1)	35 (1.9)	35.1 (1.2)	35.1 (1.5)	34.9 (1.5)
Insufficient temp. monitoring first 12 h in %	0	0	3	0	1	9	0	*NA*	0	0	2
Over- or underooling in %	16	25	38	39	29	0	27	*NA*	33	26	29
Passive cooling in %	1	0	29	31	100	9	2	0	0	12	30
Rewarming per hour, mead (sd)	*NA*	*NA*	*NA*	*NA*	*NA*	0.2 (0.1)	0.3 (0.1)	*NA*	0.2 (0.1)	*NA*	*NA*
Rewarming > 0.5 °C in %	*NA*	*NA*	*NA*	*NA*	*NA*	0	0	*NA*	0	*NA*	*NA*
cUS not performed on admission in %	4	38	13	4	28	9	32	36	12	47	22
MRI not performed between day 5–14 in %	7	0	9	5	35	0	23	50	3	19	17
Hypotension in %	58	54	89	65	81	45	54	45	88	32	67
Seizures in %	43	43	24	42	37	36	22	14	53	41	36
Coagulopathy in %	59	50	43	15	43	18	44	23	35	15	40
Infection at any time in %	1	7	3	15	16	0	7	9	3	3	7
PPHN in %	14	28	14	27	20	9	12	9	21	24	18
Died during primary hospitalization in %	16	26	27	27	12	0	5	18	15	9	16

*cUS* cerebral ultrasound, *h* hours, *MRI* magnetic resonance imaging, *NA* evaluation not performed as data deemed unreliable, *PPHN* persistent pulmonary hypertension of the newborn, *sd* standard deviation.

### Short-term outcomes

Of the 449 cooled neonates, 377 (84%) were discharged alive from the units. Unit-specific data on short-term outcomes are depicted in Table 2. Arterial hypotension was recorded in 32–89% of cases. Clinical or subclinical seizures were recorded in 14–53%. Infection rate varied between 0 and 16%. Neonatal deaths did not occur in unit 6 (of the 11 included neonates, 7 were recorded with 2-year follow-up and 4 with discharge data confirming survival) and reached up to 27% in units 2 and 3. More details on unit-specific outcome have recently been published by Grass et al. [21].

### Association between process deviations and short-term outcome

**Fig. 3:**
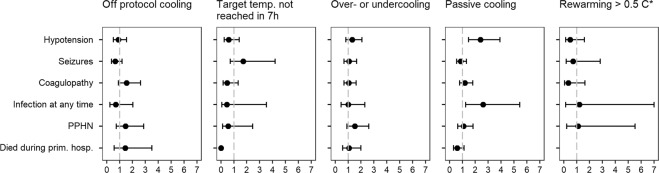
Adjusted odds ratios with 95% confidence intervals to reveal possible associations between process deviations and short-term outcome. Symbol (*) shows analysis of survivors only. Target temp. not reached in 7 h: none of the infants cooled >7 h died.

We performed an adjusted logistic regression to test possible overall associations between observed process deviations and short-term outcomes. Figure 3 displays that neonates experiencing any of the protocol deviations did not have noticeably higher odds for adverse short-term outcome, except that infants that were cooled passively had higher odds for hypotension or infection and there was a tendency toward more PPHN for overcooling or undercooling.

## Discussion

In this population-based survey of 570 neonates with HIE that were treated with TH, we display large unit-to-unit variations in deviations to the commonly agreed standardized Swiss protocol for TH (SSP). Between units, 9–36% neonates received TH without fulfilling the inclusion criteria (21% in total). These neonates were excluded from further analysis to avoid selection bias. All of the remaining 449 neonates had moderate-to-severe HIE. Compared between units, 3–10% did not reach target temperature within the foreseen timeframe and 0–38% experienced overcooling or undercooling. Three units performed the rewarming period generally according to the protocol, whereas the other units could not be analyzed due to incomplete temperature recording. None of the observable protocol deviations were associated with adverse short-term outcome.

This study is largely based on the SSP, for TH. According to Wassink et al., recent studies suggest that current protocols for TH are near-optimal, and that the key to better neurodevelopmental outcomes is earlier diagnosis and initiation of TH after birth [6]. The current protocols mentioned match the SSP which is why we believe that this protocol is currently not in need of major revision. However, earlier diagnosis of HIE may well be an area for potential improvement in Swiss units. Our baseline characteristics reveal considerable variation in pregnancy complications, delivery sentinel events, delivery mode, and physiological measures during delivery. As events during birth may affect infant outcome [22], analyzing whether the displayed variation between units is unwarranted and therefore avoidable may lead to opportunities for earlier diagnosis and/or prevention of HIE.

Another area for potential improvement is earlier initiation of TH. Thorensen et al. showed that children in which TH was started before 3 h of age had significantly better psychomotor developmental index scores at 18–24 months of age than those who were cooled after three hours postnatal age [23]. In this study, mean time to reach target temperature ranges from 3.5 to 4.5 h after birth between units. As 30 min are generally required to reach the target temperature, earlier initiation or faster induction could be another focus for improvement of TH management in Switzerland. With an outborn rate of 76% among the Swiss neonates with asphyxia, achieving earlier initiation is difficult but could be achieved by initiating active cooling during transport [24]. We also plan an in-depth analysis of the Swiss data on whether earlier initiation or faster induction of TH can lead to improved outcome.

To date, clear evidence on how strict a temperature range of 33.0–34.0 °C needs to be maintained during TH is not given [11]. Consistent clinical and preclinical findings however suggest that a relatively broad range of cooling temperatures is beneficial for the brain after HIE, and that it should not be necessary to reduce core temperatures by more than ~3.5 °C, i.e., the targeted 33.5 °C that was shown to be effective in clinical trials [6, 25]. We therefore believe that some of the Swiss units have an improvement potential by addressing measures to avoid over- and undercooling. Some of this potential may have already been addressed. When the original SSP was agreed upon before the onset of this study, there was no consensus sought regarding active versus passive cooling. However a publication of our group in 2015 revealed greater temperature stability, i.e., less temperature variability within the target range, for infants cooled actively [11]. Afterward, all remaining units cooling passively gradually changed to active cooling, including Unit 5 that switched in 2019, after the end of this study’s recruiting period. Passive cooling showed higher odds for infection and arterial hypotension in our cohort. As passive cooling was performed to a largest part by unit 5 and this unit also had the highest rate of infection (Table 2), we interpret these results as an effect of unit variation in short-term outcome as recently published [21] rather than as an association between passive cooling and the occurrence of infection or hypotension.

There is no human data available regarding the optimal rate of rewarming [26]. Rebound seizures have been described in human neonates [27] and animal newborn models of HIE injury [28] even with a rewarming rate of 0.5° per hour. Animal data suggest that slow rewarming may improve neurodevelopmental outcome by not reversing the effect of neuroprotection through the release and accumulation of excitatory neurotransmitters [29]. Recent experimental data suggest that the overall duration of cooling is the critical determinant of outcome rather than the rate of rewarming [26]. However, in neonates, Mitra et al. showed that mitochondrial injury and low cerebral metabolic rate in neonatal brain with moderate to severe ischemic injury persists even after TH during the rewarming period in comparison with neonates with evidence of mild brain injury [30]. This underlines that the rewarming period is a vulnerable phase in neonates with HIE after TH and slow rewarming might be safer until further data are available. Apart from those units that display rewarming according to the protocol, we therefore believe that better monitoring and servo-controlled devices are the key for the remaining units to ensure slow rewarming.

Cranial ultrasound (cUS) studies emphasize the importance of cUS in neonates with HIE [31]. cUS immediately after birth can demonstrate congenital structural cerebral abnormalities, fetal brain injury, and detect abnormalities due to other causes of neonatal encephalopathy, such as hypoplastic corpus callosum in cases of nonketotic hyperglycinemia, germinolytic cysts in mitochondrial or peroxismal disorders, and severe white matter echogenicity in molybdenum cofactor deficiency, also referred to as “HIE-mimics” [26, 31, 32]. In our study, the rate at which cUS was performed on the day of admission varied strongly. This may reflect that cUS is performed by neonatologists in some units, whereas radiologists perform them in others and imaging is restricted to daytime. Alternatively, it may reflect the varying degree of importance attributed to early cUS imaging by clinicians. We are currently organizing regular cUS courses to better explain the rationale of early cUS imaging aiming to increase rates further.

MRI is the optimum technique to detect perinatally acquired cerebral lesions, and the pattern and severity of the lesions provide a reliable guide to prognosis of neurodevelopmental outcome up to childhood [17, 33, 34]. A recent prospective multicenter cohort study showed that proton magnetic spectroscopy resonance biomarkers (MRS) independently gave substantial improvement in prognostic accuracy over available clinical measures and conventional MRI scoring in cooled neonates. Proton MRS is hence among the most powerful predictors for outcomes in neonates with HIE [15, 35]. By emphasizing the importance of imaging MRI in our regular register meetings, we hope to raise the awareness of the potential for both imaging modalities.

The short-term outcomes reported in our study are discussed in detail in a recent publication [21]. It summarizes the difficulty in comparing outcomes between units that have different approaches for diagnosing arterial hypotension or different degrees of expertise in detecting seizures. In comparison with United Kingdom (UK) TOBY Cooling Register cohort and a Dutch cohort, our overall outcomes are comparable or lower: [9, 36]. The overall period prevalence of HIE requiring TH is ca. 0.8 per 1000 livebirths in Switzerland and thus lower than the 1–1.5 estimated for the UK. Mortality was 32% in the Dutch, 20% in the UK TOBY, and 16% in our cohort; infections were 19% in the Dutch, 21% in the UK TOBY, and 7% in our cohort; arterial hypotension was 81% in the Dutch, 40% in the UK TOBY, and 67% in our cohort; seizures were 44% in the UK TOBY, and 36% in our cohort; coagulopathy was 44% in the Dutch, 31% in the UK TOBY, and 41% in our cohort; PPHN was 28% in the Dutch and 18% in our cohort.

The lack of observed association between process deviations and short-term outcomes in our study results either from too few neonates experiencing the deviation, a too small difference between neonates treated per protocol and those experiencing a deviation, or a limited importance in adhering to the recommended target temperatures on short-term outcomes. The first two would hint toward a limited degree of compromised quality in the Swiss cohort due to protocol deviations, the latter to a limited importance of adhering to the protocol itself. Based on the available literature listed, we favor the first interpretation.

The majority of neonates receiving TH without fulfilling the inclusion criteria had mild HIE, their cooling was initiated after 6 h or they had a lower gestational age. For neonates with mild HIE, the benefit of TH has never formally been tested, however there is growing evidence that neonates with mild HIE suffer from adverse cognitive and neuromotor outcome [37]. Although in that study, intact survival was greater after mild than moderate or severe HIE, there was no significant difference in the cognitive outcomes of neonates who had mild or moderate HIE among the survivors. Disability occurred in up to 16% in this cohort of untreated mild HIE, mostly affecting language abilities [37]. This is consistent with a recent MR imaging study which showed cerebral injuries in 54% of the neonates with mild HIE [38].

Although minor benefits have been reported for neonates with moderate HIE in whom cooling was initiated after 6 h, the effectiveness of this form of TH is yet uncertain [39, 40]. Also, a higher mortality has been reported for neonates with lower gestational age, however, there may have been selection bias [6, 9]. In short, there is no clear view on a benefit–harm ratio for neonates in which TH is considered to be used off-protocol and more research is urgently needed. An analysis of long-term outcomes at 2 and 5 years of our off-protocol cooled children is planned.

The SSP was based on the procedures developed during RCTs, in particular Azzopardi et al. [1], as RCTs are the gold standard for evidence-based methods. Implementation of highly specialized procedures from RCTs into a real-world setting may however raise issues about applicability of experimental findings and inclusion criteria [7, 41]. Different interpretation of processes and outcomes of RCTs, different views on statistical significance, and clinical importance as well as different local contexts will lead to variation between healthcare providers [42–44]. Such variations are to be expected and are usually more frequent than anticipated [45]. Our study reveals how evidence-based findings for the care of neonates with HIE were implemented in a near-population-based setting, the resulting variations between units, their lack of measurable effect on short-term outcomes and their resulting potential for quality improvement when placing those variations into the context of literature published after the SSP was agreed upon.

The strengths of our study lie in the geographical, near population-based setting, the prospective nature of data collection for quality assessment, the large number of neonates included, and the real-world nature of a cohort study versus a clinical trial. Simultaneously, as in any cohort study, we are faced with several sources of potential bias. We believe to have met the major confounders by introducing standardization and risk adjustment. However, the limited data completeness in temperature measurements may have introduced bias into the analysis of some units and disabled an analysis of rewarming after TH. With this study, we believe to have demonstrated the importance of maintaining a national registry monitoring baseline characteristics, processes and outcome as suggested by other researchers [9–11].

In conclusion, this study presents a unit-to-unit comparison of internationally agreed-upon standardized procedures for TH. We present population-based data of 449 neonates with moderate-to-severe HIE receiving TH at 10 units. Large variations between units can be observed for perinatal baseline characteristics as well as for deviations to the standardized Swiss protocol (SSP) for TH, particularly concerning reaching and maintaining target temperature during TH. None of the observed deviations however translate directly into higher odds for adverse short-time outcome. We conclude that overall, Swiss units have internationally comparable or lower proportions of adverse short-term outcome for neonates with moderate-to-severe HIE, but still display considerable improvement potential that would benefit these neonates, in particular regarding early diagnosis, early initiation, and better monitoring of TH. A long-term outcome comparison is required to substantiate these findings.

## Supplementary information

Supplement 1

## Data Availability

The datasets used and/or analyzed during the current study are available from the corresponding author on reasonable request.
