# Supplementary material for: Process variations between Swiss units treating neonates with hypoxic-ischemic encephalopathy and their effect on short-term outcome
Source: J Perinatol. 2021 Jul 21;41(12):2804–12. doi: 10.1038/s41372-021-01156-w (PMC8752440; doi:10.1038/s41372-021-01156-w)
Supplement: Supplementary file 1 — Supplement 1 [file 41372_2021_1156_MOESM1_ESM.pdf]

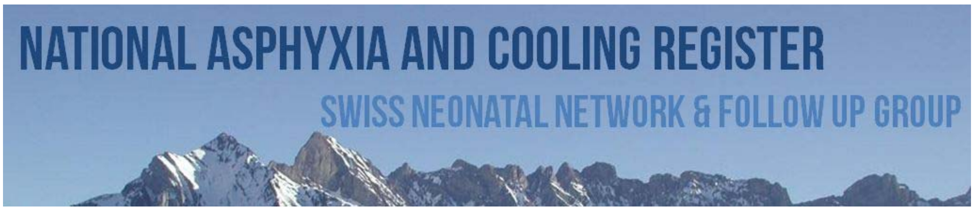

SUPPLEMENT: STANDARDIZED SWISS PROTOCOL (SSP)

1. Patient group
2. Target temperature, timing and duration of cooling
3. Clinical management
    a. Maintaining hypothermia
    b. Analgesia and sedation during hypothermia
    c. Ventilation
    d. Cardiovascular support
    e. Fluid management
    f. Sepsis
4. Neuromonitoring during hypothermia
    a. Amplitude integrated EEG (aEEG) and EEG
5. Neuroimaging
    a. Cranial ultrasound (cUS)
    b. Magnetic Resonance imaging (MRI)
6. Other investigations
7. Follow-up assessment
8. Data collection
    a. Information leaflet
    b. Daily data collection sheet

1. Patient group

Evaluate eligibility for hypothermia when resuscitation is completed and infant is stable.

Term and near term infants less than six hours old who meet the following treatment criteria (A and B) may be considered for treatment with hypothermia:

A. Infants ≥36 weeks gestation admitted to the neonatal unit, with at least two of the following:
   a. Apgar score of ≤5 at (5)10 minutes after birth
   b. Continued need for resuscitation, including endotracheal or mask ventilation, at 10 minutes after birth
   c. Acidosis within 60 minutes of birth defined as any occurrence of umbilical cord, arterial or capillary pH ≤7.00
   d. Base Deficit ≥ 16 mmol/L in umbilical cord or any blood sample (arterial, venous or capillary) within 60 minutes of birth
   e. Lactate ≥12mmol/l in umbilical cord or any blood sample (arterial, venous or capillary) within 60 minutes of birth

B. Seizures or moderate to severe encephalopathy defined by Sarnat (Stage II or III) or Thompson Score ≥7

Contraindications for therapeutic hypothermia:

- Gestational age less than 36 weeks
- More than 6 hours old
- Major congenital malformations
- Conditions requiring immediate or imminent surgery
- Pulmonary arterial hypertension refractory to treatment
- Haemorrhagic or septic shock refractory to treatment
- Severe growth restriction: BW <2000g and HC less than -2SD for GA

2. Target temperature, timing and duration of cooling

- It is important to monitor body temperature in all term infants with perinatal asphyxia to avoid hyperthermia or excessive hypothermia.
- *Continuous* rectal temperature monitoring should be initiated before cooling is started and every 15 minutes until target temperature is reached. Once target temperature is reached, *hourly* temperature recordings should be noted in the nursing chart and/or daily data recording sheets (see appendix). If temperature suddenly drops or

- During whole body cooling the target temperature is **33-34°C**. Hypothermia should be started as soon as practically possible, but within the first 6 hours after birth. Target temperature should be reached within one to two hours after initiation of hypothermia and maintained for 72 hours
- Rewarming should be performed at a rate of no more than 0.2 to 0.5°C per hour to normothermia (36.0± 0.5°C). Overshoot hyperthermia during the rewarming period should be avoided. Regular temperature control during next 48 hours after normothermia has been reached should be done to avoid hyperthermia.
- If transfer to a cooling centre is required cooling should be initiated by the referring hospital after discussion with attending neonatologist or/and the transport team, on condition that the involved clinicians are experienced in cooling.

3. Clinical management

a. Maintaining hypothermia

If target temperature cannot be achieved or maintained through passive cooling by turning off heating equipment and taking off baby blankets, clothes or hat, additional cooling equipment should be used to provide stable temperature control. *Fluctuations of temperature should be avoided and prevented.* Available whole body cooling equipments include manually adjustable thermostat-regulated Tecotherm system (Tecotherm, TSMed 200M, Tec-Com, Lübeck, Germany), the servo controlled Criticool system (MRTE, Charter Kontron, Milton Keynes, UK) and the Tecotherm servo system (Tec-Com, Lübeck, Germany)(Robertson, Kendall et al. 2010). It has been shown recently that there is less temperature variability with a servo-controlled system than with a manually adjusted system (Strohm and Azzopardi 2010). The equipment should be used according their manuals and sufficient training of the staff should be undertaken. It is essential to monitor rectal temperature continuously with hourly recordings in the daily data sheets.

b. Analgesia and sedation during cooling

Stress has adverse effect in asphyxiated infants and may affect the therapeutic effect of hypothermia. Cooling might be additional stress (Thoresen, Satas et al. 2001). Signs of distress in a cooled infant could be a consistently high heart rate above 110/min in addition to facial grimacing and irritability or metabolic acidosis. All cooled infants should be sedated. As most infants are ventilated continuous morphine infusion (10-20mcg/kg/hour) could be used and in non-ventilated infants low doses of continuous morphine (5-10mcg/kg/hour) or chloral hydrate (20-50mg/kg) could be administered. Be aware that the temperature can rapidly decrease after sedation. Hypothermia may affect the metabolism of several drugs including the sedative and analgetic medications; hence, when neurological examination is

performed, any sedative/analgetic medication should be noted. Paralysis is administered as clinically indicated.

c. Ventilation

Most cooled infants require initially mechanical ventilation. Ventilation will be managed according to the hospital policy and blood gases will guide the ventilation. Aimed are P_aCO_2 of 5-7kPa (37.5-52.5mmHg) at 33°C and P_aCO_2 of 6-8 at 37°C (45.1-60.2) body temperature and P_aO_2 of 6-10kPa (45-75mmHg).

d. Cardiovascular support

Invasive blood pressure monitoring is advised. Most infants' heart rate might be decreased and their blood pressure might increase during cooling. The common heart rate in cooled term infants will be around 100/min and mean blood pressure >38-40mmHg. Cardiovascular support should be done according to unit policy. Echocardiography should be performed as clinically indicated.

e. Fluid management

As renal function is usually impaired in infants with perinatal asphyxia initial fluid requirement should be between 40-60 ml/kg/day and further fluid management will be guided by regular blood creatinine, electrolyte, blood gases, urine output and weight. Urine output of >1ml/kg/hr should be aimed at. Hypoglycaemia and hyperglycaemia should be avoided. If oral feeds (trophic feeds) are given then with caution during cooling. Na, K, creatinine, liver enzymes and coagulation should be monitored daily during the cooling and rewarming period (see table).

f. Seizures

Seizures should be treated according to the local unit's guidelines. Hypothermia may affect the metabolism of several drugs including the anticonvulsants, hence, drug levels should be monitored closely. Seizures might reoccur during the rewarming period, therefore aEEG monitoring should be continued during the rewarming period.

g. Sepsis

As hypothermic infants might be more prone to infections, antibiotics should be given as clinically indicated. CRP, blood film and leucocytes should be done daily during the cooling and rewarming period and thereafter as clinically indicated.

4. Neuromonitoring during hypothermia

a. Amplitude integrated EEG (see appendix) and EEG

Brain function should be monitored in all infants with HIE. aEEG may help to decide whether an infant should be treated with therapeutic hypothermia. However, cooling should not be delayed until aEEG is available. The aEEG findings should be documented in the patient' notes according to the classification of de Vries et al (see appendix)(de Vries and Hellstrom-Westas 2005). In cooled infants aEEG should be performed continuously during the cooling and rewarming period. Formal EEG should be done at any time if clinical or electrographical seizures are seen and routinely during the cooling period and before discharge. If any EEG abnormalities are seen then it should be repeated before discharge.

5. Neuroimaging

a. Cranial ultrasound

cUS should be performed on admission to exclude structural brain malformation, to document evidence of long standing or more recently established injury and to detect abnormalities characteristic of non-HIE causes of encephalopathy such as a hypoplastic corpus callosum suggesting diagnosis of non-ketotic hyperglycinaemia and germinolytic cysts suggesting mitochondrial or peroxisomal disorders or congenital infections.

Daily cUS will demonstrate the evolution of brain injury. Doppler ultrasound including pulsatility index (normal values between 0.65-0.85) gives useful prognostic information. Values below 0.55 occur in severe HIE usually between day 2 and 4 after birth and are associated with poor outcome (Levene, Fenton et al. 1989). Abnormal values within the first six hours after birth suggest an insult either intrapartum or 1-2 days prior to delivery (Eken, Toet et al. 1995). cUS should be performed beyond the first week of life and also after MR is done as it will show further evolution of the injury.

b. Magnetic resonance imaging/ spectroscopy

MRI provides details of brain lesions characteristic of perinatal hypoxic-ischaemic injury, the lesions can be graded and related to outcome (Rutherford, Ramenghi et al. 2010). MRI should be performed between day 5-14 and include, T2 and T1 weighted images, diffusion weighted imaging (ADC map) and ^{1}H Magnetic Resonance Spectroscopy (MRS) (thalamic and white matter voxel) (Thayyil, Chandrasekaran et al. 2010). If early MRI (<72 hours after birth) is done then it should be repeated at a later stage (5-14 days) as conventional MR/DWI might underestimate the severity of injury at early age.

c. Evoked potentials

Evoked potentials should be considered before discharge

6. Other investigations

Investigations for metabolic or genetic disorders if unusual clinical neurological course, unusual pattern of injury on MRI scan and/or normal looking MR scan in face of ongoing neurological problems. Consider further investigations such as ammonia, repeat lactate, uric acids, amino acids, amino and organic acids (urine), urine for ketone and reducing substances, creatinine kinase and chromosomes. Look for specific disorders such as sulfite oxidase deficiency, non-ketotic hyperglycinaemia, biotinidase deficiency, peroxisomal or mitochondrial disorders.

7. Follow-up assessment

Follow up according to unit's policy but at least

a. at 2 year of age

Bayley II PDI and MDI, neurological examination, CP, gross motor function, visual

b. at 5 years of age

K-ABC II, neurological exam, CP, "Lageuntersuchung nach Largo", visual exam, hearing exam.

8. Data Forms

- **a.** Information leaflet to be given to parents during hospitalisation
- **b.** Daily work flow forms to be filled in either daily or in retrospect, data entry into database directly online
- **c.** Posters with cooling criteria might be put on labour ward and the neonatal unit

Investigations	0-24 hrs	25-48hrs	49-72hrs	72-80hrs	comment
CRP, Lcdiff, Hb, Hkt, coagulation*	X	X	X	X	*Coagulation screening on day 1 and afterwards as clinically indicated
Blood gas, Lactate, Na, K, TBG, Creatinine, ALT, Mg	X	X	X	X	Liver enzyme repeated as clinically indicated
aEEG	X	X	X	X	Continuous monitoring
cUS with Doppler	X	X	X	X	At 7 and 14 days of age, then two weekly until discharge
Neurological assessment (Sarnat or/and Thompson)	X	X	X	X	Before discharge
EEG	If seizures on aEEG	If seizures on aEEG	If seizures on aEEG	If seizures on aEEG	Day 4-7
MRI/MRS					Day 5-14
Daily data sheet	X	X	X	X	
Placental histology	X				should be sent for histology

Appendix

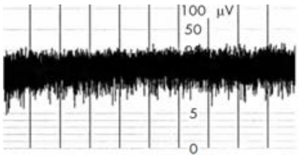

1. Continuous normal voltage pattern (CNV): continuous activity with lower (minimum) amplitude around (5) to 7 to 10uV and maximum amplitude around 10 to 25 (to 50) uV

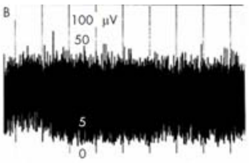

2. Discontinuous normal voltage pattern (DNV): discontinuous background, with variable minimum amplitude, but less than 5uV and maximum amplitude greater than 10uV

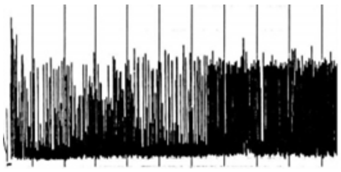

3. Burst suppression (BS): discontinuous background with minimum amplitude without variability at 0 to 1 (2) uV and bursts with amplitude greater than 25uV

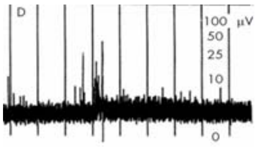

4. Continuous low voltage (CLV): continuous background pattern of extremely low voltage (around or less than 5uV)

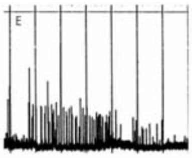

5. Inactive flat trace (FT): mainly inactive (isoelectric tracing) background less than 5uV

Appendix 1. aEEG Classification (Ref)

**Thompson Score**				
**Sign**	**0**	**1**	**2**	**3**
Tone	Normal	Hypertone	Hypotone	Flaccid
LOC	Normal	Hyperalert, stare	Lethargic	Comatose
Fits	Normal	Infrequent <3/day	Frequent >2/day	
Posture	Normal	Fisting, cycling	Strong distal flexion	Decerebrate
Moro	Normal	Partial	Absent	
Grasp	Normal	Poor	Absent	
Suck	Normal	Poor	Absent/ bites	
Respiration	Normal	Hyperventilation	Brief apnoea	Apnoeic
Fontanelle	Normal	Full, not tense	Tense	

Appendix 2. Thompson Score (Thompson, Puterman et al. 1997). LOC, level of consciousness

	**Stage 1**	**Stage 2**	**Stage 3**
**Level of consciousness**	Alert	Lethargic or obtunded	Stuporous
**Neuromuscular Control**			
Muscle tone	Normal	Mild hypotonia	Flaccid
Posture	Mild distal flexion	Strong distal flexion	Intermittent decerebration
Stretch reflexes	Overactive	Overactive	Decreased or absent
Segmental myoclonus	present	present	absent
**Complex Reflexes**			
Suck	Weak	Weak or absent	Absent
Moro	Strong; low threshold	Weak; high threshold	Absent
Oculovestibular	Normal	Overactive	Weak or absent
Tonic neck	slight	strong	absent
**Autonomic function**			
Pupils	Mydriasis	Miosis	Variable; unequal, poor light reflex
Heart Rate	Tachycardia	Bradycardia	Variable
Salivary Secretions	Sparse	Profuse	Variable
GI Motility	Normal or decreased	Increased; diarrhoe	variable
**Seizures**	none	common	uncommon

Appendix 3. Sarnat Score (Sarnat and Sarnat 1976)

References

de Vries, L. S. and L. Hellstrom-Westas (2005). "Role of cerebral function monitoring in the newborn." Arch Dis Child Fetal Neonatal Ed **90**(3): F201-207.

Eken, P., M. C. Toet, et al. (1995). "Predictive value of early neuroimaging, pulsed Doppler and neurophysiology in full term infants with hypoxic-ischaemic encephalopathy." Arch Dis Child Fetal Neonatal Ed **73**(2): F75-80.

Levene, M. I., A. C. Fenton, et al. (1989). "Severe birth asphyxia and abnormal cerebral blood-flow velocity." Dev Med Child Neurol **31**(4): 427-434.

Robertson, N. J., G. S. Kendall, et al. (2010). "Techniques for therapeutic hypothermia during transport and in hospital for perinatal asphyxial encephalopathy." Semin Fetal Neonatal Med **15**(5): 276-286.

Rutherford, M., L. A. Ramenghi, et al. (2010). "Assessment of brain tissue injury after moderate hypothermia in neonates with hypoxic-ischaemic encephalopathy: a nested substudy of a randomised controlled trial." Lancet Neurol **9**(1): 39-45.

Sarnat, H. B. and M. S. Sarnat (1976). "Neonatal encephalopathy following fetal distress. A clinical and electroencephalographic study." Arch Neurol **33**(10): 696-705.

Strohm, B. and D. Azzopardi (2010). "Temperature control during therapeutic moderate whole-body hypothermia for neonatal encephalopathy." Arch Dis Child Fetal Neonatal Ed **95**(5): F373-375.

Thayyil, S., M. Chandrasekaran, et al. (2010). "Cerebral magnetic resonance biomarkers in neonatal encephalopathy: a meta-analysis." Pediatrics **125**(2): e382-395.

Thompson, C. M., A. S. Puterman, et al. (1997). "The value of a scoring system for hypoxic ischaemic encephalopathy in predicting neurodevelopmental outcome." Acta Paediatr **86**(7): 757-761.

Thoresen, M., S. Satas, et al. (2001). "Twenty-four hours of mild hypothermia in unsedated newborn pigs starting after a severe global hypoxic-ischemic insult is not neuroprotective." Pediatr Res **50**(3): 405-411.
